# A New Surface Charge Neutralizing Nano-Adjuvant to Potentiate Polymyxins in Killing Mcr-1 Mediated Drug-Resistant *Escherichia coli*

**DOI:** 10.3390/pharmaceutics13020250

**Published:** 2021-02-11

**Authors:** Hyejin Cho, Atanu Naskar, Sohee Lee, Semi Kim, Kwang-Sun Kim

**Affiliations:** 1 Department of Chemistry and Chemistry Institute for Functional Materials, Pusan National University, Busan 46241, Korea; chj9512@pusan.ac.kr (H.C.); atanunaskar@pusan.ac.kr (A.N.); kin5497170@pusan.ac.kr (S.L.); 2 Immunotherapy Research Center, Korea Research Institute of Bioscience and Biotechnology, Daejeon 34141, Korea; semikim@kribb.re.kr

**Keywords:** nano-antibiotics, black phosphorus, polymyxin-resistant Gram-negative bacteria, charge neutralization mechanism, Mcr-1

## Abstract

Resistance to polymyxins when treating multidrug-resistant (MDR) Gram-negative bacterial infections limit therapeutic options. Here, we report the synthesis of a nickel (Ni) doped Zinc oxide (NZO) combined with black phosphorus (BP) (NZB) nanocomposite and its synergistic action with polymyxin B (PolB) against polymyxin-resistant *Escherichia coli* harboring mobilized colistin resistance (*mcr-1*) gene. NZB and PolB combination therapy expressed a specific and strong synergy against Mcr-1 expressing *E. coli* cells. The underlying mechanism of the synergy is the charge neutralization of the *E. coli* cell surface by NZB, resulting in a more feasible incorporation of PolB to *E. coli*. The synergistic concentration of NZB with PolB was proved biocompatible. Thus, the NZB is the first biocompatible nano-adjuvant to polymyxins against polymyxin-resistant *E. coli* cells, recognizing the physical status of bacteria instead of known adjuvants targeting cellular gene products. Therefore, NZB has the potential to revive polymyxins as leading last-resort antibiotics to combat polymyxin-resistant Gram-negative bacterial infections.

## 1. Introduction

Infections caused by multidrug-resistant (MDR) Gram-negative bacteria pose a serious global mortality burden and an increased death rate. In healthcare environments, Gram-negative bacteria cause infections including pneumonia, bloodstream infections, wound or surgical site infections, and meningitis [1]. In particular, patients in the intensive care unit (ICU) are exposed to a high risk of such infections. The rapid resistance of Gram-negative bacteria to currently available antibiotics and their innate ability to survive environmental pressure is of great concern. Horizontal gene transfer has been considered a major strategy to exchange genetic materials from one bacterium to another to disseminate drug-resistance [2]. However, the lack of effective antimicrobial agents to combat MDR pathogens has led to the resurgence of last-resort antibiotics including polymyxins (polymyxin B and colistin) to control MDR Gram-negative bacterial infections [3]. Researchers are also trying in different ways such as detection of bacteria which can be utilized to kill bacterial cells [4,5,6].

Last-resort polymyxins have been revived in the treatment of MDR Gram-negative bacterial infections even though the risk of polymyxin resistance by EptA and ArnT, which modify lipid A with a phosphoethanolamine (pEtN) residue and 4-amino-4-deoxy-l-arabinose (l-Ara4N), respectively, was suggested [7]. Fortunately, until 2015, strains with plasmid encoded resistance to polymyxins have not been identified. However, the emergence of transferable polymyxin resistance by the mobilized colistin resistance (*mcr-1*) gene, which encodes a protein Mcr-1, a family member of EptA [7,8], jeopardized polymyxin resurgence as a last-resort defense against MDR bacterial infections [9,10]. Lipid A modification on the lipopolysaccharide (LPS) of the cell surface with EptA reduces the overall net-negative charge of the outer membrane of some Gram-negative bacteria [8]. This confers resistance to positive charged polymyxins through positive charge repulsion between the outer membrane surface of the cell and polymyxins, resulting in reduced polymyxin incorporation into cells. Therefore, development of a new generation of antibiotics, inhibitors, alternative materials, or therapeutics to control such resistance mechanisms is necessary. 

A novel colistin adjuvant that inhibits ArnT, which catalyzes the last committed step of lipid A aminoarabinosylation and is associated with a positive charge increase in cells [7], was identified using in silico screening [11]. Therefore, the strategy appears to be a promising candidate for lead optimization in the development of colistin resistance inhibitors. However, target activity was solely dependent on target gene expression, resulting in a possible occurrence of bacterial resistance by multiple uses through new ways to survive antibiotic pressure strategies [12]. Therefore, new materials that are not associated with targeting specific cellular gene products need to be developed, rather than recognizing the physical cellular status. 

Nano-antibacterials using nanotechnology have progressively emerged owing to its various advantages such as limited immunogenicity and cytotoxicity, favorable drug release profiles, prolonged circulation, extended half-lives, and targeting ability of specific cells and tissues [13,14,15]. More importantly, nano-antibiotics prefer multi-target approaches rather than the single target approach generally used by antibiotics [16]. Hence, the chance of bacterial resistance against nano-antibiotics may be less compared to that against antibiotics. Additionally, the disadvantages of other alternative antibacterial therapies (antibiotic combination and drug repurposing) can be addressed with nano-antibacterials [13]. Owing to the various advantages, it is imperative that there are increasing attempts to use the nano-antibacterials for controlling the bacterial infections and preventing antibiotic resistance by synergistic therapy of nanomaterials with lower dosage of last-resort antibiotics against Gram-negative bacterial infections.

Among such nano-antibacterials, zinc oxide (ZnO) is a well-known agent that disrupts the bacterial cell membrane and inhibits growth [17]. It is also recognized as a safe material by the US Food and Drug Administration (21CFR182.8991) [18]. Additional advantages include a UV-blocking property, white appearance, and low cost. Moreover, ZnO can induce antibacterial activity by several mechanisms such as bacterial cell membrane disruption, ROS (Reactive Oxygen Species) production, leakage of intracellular contents, and others. [13]. It can also act synergistically with other nanomaterials resulting in excellent antibacterial activity [19].

Black phosphorus (BP), as a promising post-graphene 2D material, has emerged in biomedical science because of its favorable properties in various biomedical applications such as photothermal therapy, drug delivery, biosensing, and bioimaging [20,21]. BP also acts as an effective antibacterial agent that disrupts the cell membrane of bacteria [16]. Moreover, BP nanosheets fabricated via other positive charged metal ion intercalation exhibited a high negative charge and showed high reactivity to positive charged molecules [22]. Recently, our group fabricated a non-toxic Ni doped ZnO nanostructure, which specifically kill Gram-negative bacteria by supplementary ROS production [17]. Furthermore, both BP and ZnO are non-toxic and biocompatible when they are used at a low dosage [17,23]. Notably, polymyxin B (PolB) also has shown its synergistic capability with nanoparticles for antibacterial activity [24]. Therefore, a Ni doped ZnO (NZO), a previously reported to show comparatively more effective against Gram-negative bacteria assessed by zone of inhibition method [17], combined with a BP nanocomposite should synergistically act with PolB for excellent antibacterial activity against MDR Gram-negative bacteria.

Therefore, the aim of this study is to find a nano-adjuvant to potentiate polymyxins in killing Mcr-1 mediated drug-resistant *Escherichia coli*. It has been known that Mcr-1 action modified the bacterial surface charge to be more positive compared to non-Mcr-1 expressing bacteria [25]. Therefore, we hypothesized that nanoparticles (NPs) of high specificity to Gram-negative bacteria with positive charge neutralizing activity will specifically be used as potential targeting materials for Mcr-1 associated polymyxin-resistant *E. coli* cells. To test our hypothesis, a Ni doped ZnO combined with a BP (NZB) nanocomposite was designed and prepared for use as a synergistic nano-antibacterial agent with PolB to specifically target Mcr-1 expressing *E. coli*.

## 2. Experimental

### 2.1. Synthesis of Ni Doped ZnO Nanoparticles (NZO)

The NZO, which possesses 5 atomic percent (at%) (with respect to Zn^2+^) ZnO-based NPs, was synthesized as previously described in our previous work [17]. 

### 2.2. BP Nanosheets and Ni Doped ZnO-BP (NZB) Nanocomposite Syntheses

#### 2.2.1. BP Nanosheet Synthesis

A previously reported method [26] was used to synthesize BP nanosheets using bulk BP crystals. The ultrasonication-assisted aqueous exfoliation process was used for this synthesis process. Initially, 2 g of NaOH was added to 60 mL of *N*-methyl-2-pyrrolidone (NMP) solvent for 5 min under water bath sonication. Subsequently, the supernatant was collected after centrifugation. Thereafter, bulk BP crystals (25 mg) were added to the NMP-containing saturated NaOH solution. The resulting suspension was ultrasonicated for 8 h with the using an ice bath ultrasonicator while maintaining the bath temperature below 20 °C. Thereafter, unexfoliated BP crystals were separated by centrifugation at 2000 rpm for 15 min. The supernatant was subsequently collected and centrifuged again at 13,000 rpm for 10 min. Finally, the BP nanosheets collected from the centrifugation process was dispersed in water and stored at 4 °C for further use. 

#### 2.2.2. NZB Nanocomposite Syntheses

Five milliliters of as-prepared BP nanosheets of (2 mg·mL^−1^) were added to 100 mg of as-synthesized NZO in 40 mL of deionized water (DW) and was continuously stirred for 10 min. Thereafter, the mixture was ultrasonicated for 10 min. The mixture was subsequently stored for 6 h with continuous stirring. Thereafter, the sample was collected by centrifugation and vacuum dried at 60 °C for 6 h.

### 2.3. Preparation of the Mcr-1 Expression Plasmid

To prepare an Mcr-1 protein expression plasmid, DNA sequences of the Mcr-1 encoding gene (*mcr-1*) were adapted from the NCBI protein database (GenBank ID: ASK04346.1) and codon optimized using GenSmart™ Codon Optimization (ver. Beta 1.0; GenScript, Piscataway, NJ, USA). The resulting DNA sequences (Table S1) were synthesized and subcloned into pQE60 (Qiagen, Valencia, CA, USA) linearized with *Bam*HI/*Bgl*II. The subcloned DNA was confirmed by DNA sequencing analysis using primer pQE60-F and -R. The resulting plasmid was transformed into the BW25113 strain for further experiments. 

### 2.4. Characterization

#### 2.4.1. Material Properties

X-ray diffractometer (D8 Advance with the DAVINCI design, Bruker, Billerica, MA, USA) equipped with a nickel-filtered Cu Kα radiation source (λ = 1.5406 Å) was used to measure X-ray diffraction (XRD) patterns of the as-prepared ZO, NZO, and NZB samples in the 2θ range of 5–80°. The microstructure of the representative NZB sample was evaluated through transmission electron microscopy (TEM; Bruker Nano GmbH, Berlin, Germany). Carbon-coated 300 mesh Cu grids were used in this regard. Furthermore, binding energies and chemical states of the elements in the representative NZB sample were evaluated using an Axis Supra scanning X-ray photoelectron spectroscopy (XPS) microprobe surface analysis system (Kratos Analytical, Manchester, UK) in the binding energy range from 200 to 1200 eV. The C 1s peak position at 284.5 eV was used as the binding energy reference.

#### 2.4.2. Zeta Potential Measurement

Zeta (ζ) potential (millivolts, mV), determined from electrophoretic mobility μ at 25 °C in 1 mM NaCl, using Smoluchowski’s equation:

ζ = μη/ε,

where η is the medium viscosity and ε the medium dielectric constant [27], of BP, NZO, and NZB or *E. coli* cell suspensions in DW were measured with a Nanopartica SZ-100 (Horiba Scientific) according to the manufacturer’s instructions. *E. coli* cells of BW25113 and NCCP16283 were cultured overnight with and without treatment of sub-minimum inhibitory concentration (MIC) concentration of NZB (125 μg·mL^−1^), were diluted to OD_600_ = 0.5. Thereafter, cell pellets were obtained by centrifugation at 12,000 rpm for 2 min, washed three times with phosphate-buffered saline (PBS), and resuspended in 1 mL of DW to measure the zeta potential. Zeta potential values shown for individual samples are the means of at least five measurements ± standard deviation (*p* < 0.05). 

#### 2.4.3. Bacterial Strains, Growth, and Plasmids

Strains and plasmids used in this study are listed in Table S2. Strains were grown in Luria-Bertani (LB) broth or on agar plates with or without antibiotics at 37 °C for 16 h. The Keio (*E. coli* K-12 in-frame, single-gene knockout mutants) collection [28] was used after validating the deletion of the target gene using colony PCR with k1 [29] and gene specific oligonucleotides (Table S2) with Quick-Taq HS Dye Mix (Toyobo, Osaka, Japan) according to the vendor’s protocol.

#### 2.4.4. Expression Analysis of Mcr-1 Protein

Mcr-1 protein expression from the pQE60-*mcr-1* plasmid was characterized by SDS-PAGE gel electrophoresis and Western blotting. Briefly, Mcr-1 protein expression was firstly electrophoresed on 12% Mini-PROTEAN Stain-free TGX Precast gels (Bio-Rad, Hercules, CA, USA). Western blotting for Mcr-1 was performed using antibodies against histidine tag (Sino Biological, Wayne, PA, USA) and Clarity™ Western ECL substrate (Bio-Rad, CA, USA). Image analysis was performed using ChemiDocMP (Bio-Rad, Hercules, CA, USA) and Image Lab (ver.5.2.1, Bio-Rad, Hercules, CA, USA). One of the representatives from *n* = 3 was shown.

#### 2.4.5. Preparation of Bacterial Cells

Cells used for antibacterial activities and morphological characterization were prepared as follows. Initially, bacterial colonies were restreaked on LB agar plates with or without antibiotics and were resuspended with DW to an optical density of 0.5 McFarland turbidity standard using a Sensititre™ Nephelometer (Thermo Fisher Scientific, Waltham, MA, USA). Individual cells were inoculated into Sensititre™ Cation adjusted Mueller-Hinton broth (Thermo Fisher Scientific, Waltham, MA, USA) with 1000-fold dilution and were grown further at 37 °C for 16 h without shaking [16,17]. 

#### 2.4.6. Evaluation of Antibacterial Activity

Proposed antibacterial samples with controls were divided into four groups and prepared at indicated concentration ranges: (1) NFW (control), (2) NZB (0–250 μg·mL^−1^), (3) PolB (0–3.2 μg·mL^−1^; Sigma-Aldrich, Missouri, MO, USA), and (4) NZB with PolB. To measure the antibacterial activity of proposed antibiotics, prepared bacterial cells (Section 2.4.5.) were aliquoted into a 96-well plate containing the control, PolB, NZB, and NZB/PolB and cultured at 37 °C for 16 h without shaking. The minimum inhibitory concentration (MIC) and synergistic activity as the fractional inhibitory concentration index/indices (FICI) were determined using checkerboard assays and were interpreted as described [30]. At least three biological replicates were performed for evaluation of synergy of NZB with PolB. 

#### 2.4.7. Morphological Characterization of Bacteria

The morphological analysis of cells was examined [31]. Thus, four samples from checkerboard assays (Section 2.4.6) [control, sub-MIC of PolB, NZB, and NZB/PolB] were collected by centrifugation at 10,000× *g* for 1 min. The resulting cell pellets were resuspended in 500 µL PBS containing 2% formaldehyde and 1% glutaraldehyde, and centrifuged for 5 min. The final cell pellet was washed twice and resuspended in 1 mL of DW. Five microliter aliquots were collected from the suspension and deposited on a silicon wafer (5 × 5 mm, Namkang Hi-Tech Co., Ltd., Seongnam, Korea) to dry at room temperature. VEGA3, a versatile tungsten thermionic emission scanning electron microscopy (SEM) system (TESCAN, Fuveau, France), was used to study the dried wafer according to the manufacturer’s protocol.

#### 2.4.8. Reactive Oxygen Species (ROS) Production Determination

The ROS production by *E. coli* KS7000 and KS8000 after treatment with samples was evaluated based on a previous report with minor modifications [17]. Briefly, overnight cultured bacterial cells were adjusted to 0.5 McFarland turbidity in PBS buffer using Sensititre™ Nephelometer (Thermo Scientific, MA, USA). Then, the cell suspension (1 mL) was treated with different nanocomposites in the presence of 2′,7′-dichlorodihydrofluorescin diacetate (DCFH-DA) (Sigma-Aldrich, Missouri, MO, USA) at a final concentration of 30 µM in PBS buffer. The suspension (200 µL) was aliquoted in 96-well plate and incubated at 37 °C with vigorous shaking (500 rpm) using Incu-Mixer™ MP (Benchmark Scientific, Sayrevile, NJ, USA) for 1 h. Then, fluorescence intensity with an excitation and emission wavelengths of 485 and 520 nm, respectively, was measured by FLUOstar Omega (BMG Labtech GmbH, Ortenber, Germany). Untreated bacterial culture was used as a negative control. MARS Data Analysis software (ver. 3.02 R2, BMG Labtech GmbH, Ortenber, Germany) was used to further analyze the samples. The samples were measured in triplicate and the relative ROS production of samples treated with NPs was compared.

#### 2.4.9. Biocompatibility Assays and Morphological Change

HEK293 cells from the American Type Culture Collection (Manassas, VA, USA) were maintained in RPMI1640 with 10% of fetal bovine serum at 37 °C in 5% CO_2_. Cell viability was determined via the colorimetric WST assay (Ez-Cytox; DoGenBio, Seoul, Korea). Cells were seeded into 96-well plates at a density of 5000 cells per well and incubated for 24 h. Thereafter, cells were further incubated for 24 or 48 h in the presence of NZB sample at a concentration of 10–200 μg·mL^−1^ in 0.1% dimethyl sulfoxide, followed by cell incubation with the WST reagent (one-tenth of the medium volume). The amount of formazan dye formed was determined by measuring the absorbance at 450 nm using a spectrophotometric microplate reader (BMG LABTECH GmbH, Ortenber, Germany). The morphology of HCT-15 cells treated with the above samples was imaged using a phase-contrast microscope (Leica DM IL LED; Leica, Wetzlar, Germany) (data not shown).

## 3. Results and Discussion

### 3.1. Material Properties

#### 3.1.1. Phase Composition

**Figure 1:**
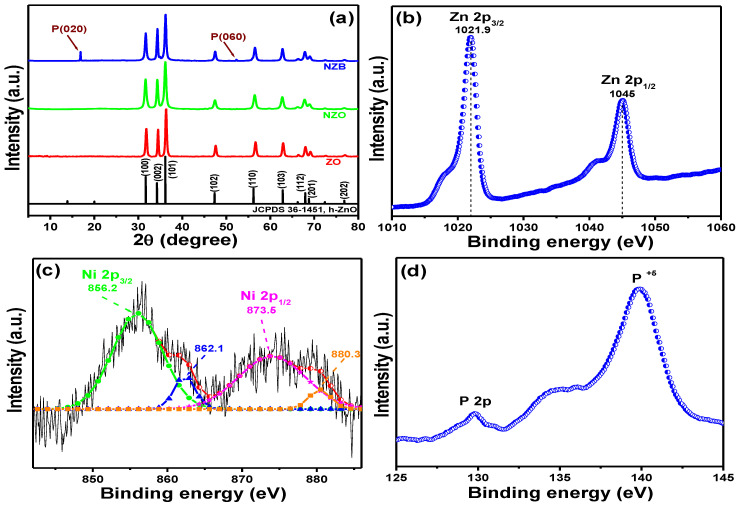
(**a**) XRD patterns of ZO, NZO (nickel (Ni) doped zinc oxide), and NZB (NZO combined with a black phosphorus nanocomposite)-samples. XPS spectral analysis of the NZB nanocomposite: (**b**) Zn *2p* peaks. (**c**) Gaussian-fitted curves of the Ni *2p* peaks. (**d**) P *2p* core peaks.

The crystalline phases of ZO, NZO, and NZB samples were evaluated using XRD and shown in Figure 1a. XRD reflection peaks of the as-synthesized ZO, NZO, and NZB were consistent with the hexagonal ZO (h-ZO) structure (JCPDS 36-1451) [17]. However, some additional peaks can be seen at approximately 16.78° and 52.38° for the NZB sample. These peaks can be attributed to the (020) and (060) lattice planes of BP [32]. These results also suggested that even after liquid ultrasonication, there was no impurity and exfoliated BP nanosheets are still in the orthorhombic system. This XRD result confirmed the successful formation of the NZB nanocomposite.

#### 3.1.2. XPS Results

The elemental composition and valence state of the representative NZB sample was measured by XPS and the spectra are depicted in Figure 1b–d, which shows the binding energy signals of Zn *2p* (Figure 1b), Ni *2p* (Figure 1c), and P *2p* (Figure 1d). For Zn *2p* (Figure 1b), two strong peaks were detected at 1021.9 and 1045.0 eV, which were assigned to the binding energies of the Zn *2p*_3/2_ and Zn *2p*_1/2_ states, respectively [16]. Moreover, the calculated binding energy difference between Zn *2p*_3/2_ and Zn *2p*_1/2_ was ~23.1 eV, confirming the valence state of zinc as Zn^2+^ [16]. Figure 1c shows the Gaussian fitted binding energy curves of Ni *2p*. Its two main peaks were centered at approximately 856.2 and 873.5 eV, representing the binding energies of the Ni *2p_3/2_* and Ni *2p_1/2_* states, respectively [17]. Similarly, the calculated energy difference between the Ni *2p_3/2_* and Ni *2p_1/2_* states was 17.3 eV, which is quite different from NiO (18.4 eV) [33]. Therefore, the above results confirm that the Ni present in NZB is different from NiO. Additionally, Figure 1d shows the binding energies of the P *2p* peak, which is in agreement with other published data [23]. Therefore, XPS data (Figure 1b–d), along with data from XRD (Figure 1a), confirmed successful formation of the NZB nanocomposite.

#### 3.1.3. Morphology and Microstructure

**Figure 2:**
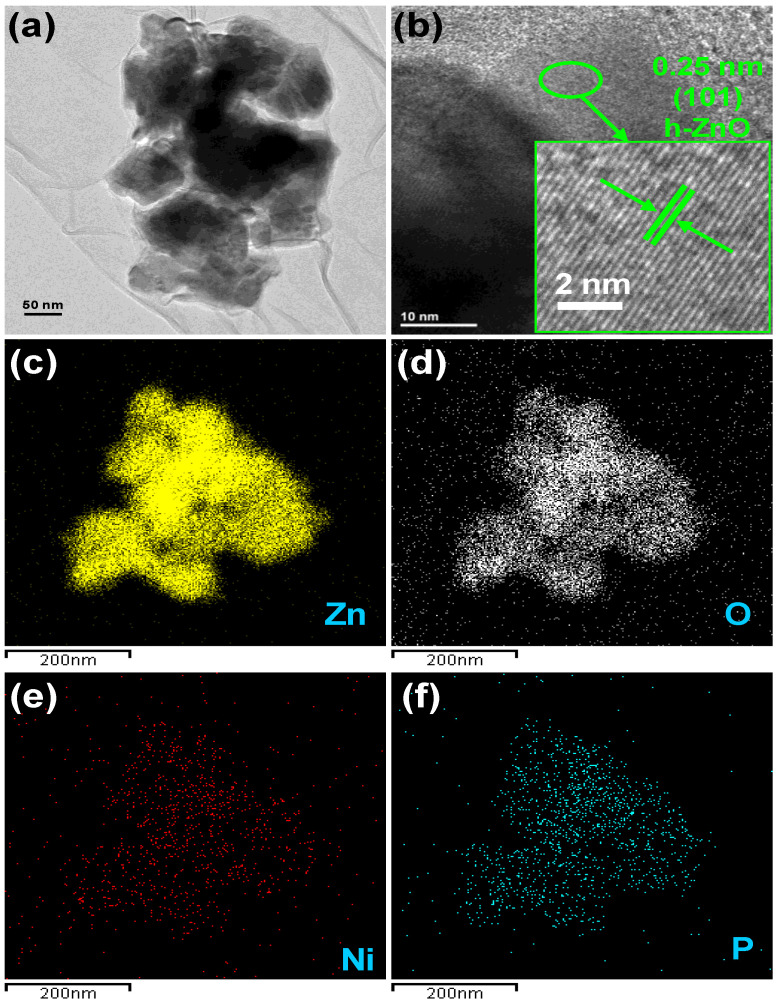
(**a**) TEM image and (**b**) HRTEM image of the NZB sample. Elemental mapping images for (**c**) zinc, (**d**) oxygen, (**e**) Ni, and (**f**) P elements of the NZB sample.

The morphology and microstructure of representative NZB is depicted in Figure 2. Figure 2a shows the TEM images of NZB nanoparticles where NZO nanoparticles were distributed on BP nanosheets. Similarly, the high-resolution TEM (HRTEM) image of NZB is shown in Figure 2b. The inset of Figure 2b shows the enlarged version from the marked region in Figure 2b. The distinct lattice fringes with interplanar distances of 0.25 can correspond to the (101) plane of ZO. Additionally, elemental mapping images of the representative NZB sample showed good distributions of Zn (Figure 2c), O (Figure 2d), Ni (Figure 2e), and P (Figure 2f) elements. Hence, TEM and HRTEM images as well as elemental mapping confirmed the successful formation of the NZB nanocomposite.

### 3.2. Antibacterial Activity 

#### 3.2.1. Evaluation of Antibacterial Activity of NZB against *E. coli* Strains

Our primary aim of NZB preparation, as mentioned earlier, is its use as a selective nano-adjuvant against polymyxin B-resistant *E. coli* strains. To this end, we need to have multiple *E. coli* strains with different features of antibiotic resistance. Initially, the minimum inhibitory concentration (MIC) of PolB against standard, NCCP isolates, and NDM-1 MDR *E. coli* strains was assessed by microbroth dilution method, a reliable method for the susceptibility of PolB against Gram-negative bacteria [34]. As shown in Table 1 and Figures S1 and S2 (Supplementary Materials), the MIC value of PolB was 0.8 μg·mL^−1^ for BW25113 with or without the control vector and NDM-1 *E. coli* strains. Thus, NDM-1 *E. coli* isolates used in this study are still susceptible to PolB in wild type cells. Meanwhile, NCCP isolates, containing the Mcr-1 expressing gene (*mcr-1*) as a mobile element [35], showed 4-times more resistance to PolB (MIC = 3.2 μg·mL^−1^) compared to the BW25113 strain (Table 1). As these isolates might have multiple resistant genes, it is difficult to see the sole Mcr-1 effect on susceptibility. Therefore, the Mcr-1 expression vector was prepared by synthesizing DNA sequences for Mcr-1 coding regions with C-terminal histidine tag after codon optimization to be maximally expressed in *E. coli* (Table S2, Figure S3) and subcloned into a plasmid pQE60, resulting in pQE60-*mcr-1* (Figure S4). The resulting plasmid was transformed into the *E. coli* BW25113 strain and used for the evaluation of the effect of Mcr-1 on PolB susceptibility. Mcr-1 expression in isopropyl β-d-1-thiogalactopyranoside-induced cells was analyzed by Western blotting using antibodies against histidine tag (Figure S4), confirming that the protein is well expressed. This strain was further confirmed by MIC assays and found that Mcr-1 protein overexpression increased resistance to PolB (MIC = 3.2 μg·mL^−1^) in NCCP isolates. This indicates that PolB resistance of NCCP isolates originated from Mcr-1 protein expression, and this system could evaluate the role of Mcr-1 on multiple treatments. Next, we further determined the antibacterial activity of nanocomposites against *E. coli* cells using MIC assays. We observed that MIC values of BP, NZO, and NZB was >500, 200, and 250 μg·mL^−1^, respectively, for all tested bacteria (Table 1**;** data not shown for BP and NZO), suggesting that NZB itself displayed weak antibacterial activity originated from NZO origin, not BP, with non-selectivity of antibiotic resistance status of strains (Table 1, Figure S2). 

#### 3.2.2. Specific Synergistic Action of NZB to PolB on Mcr-1 Expressing *E. coli* Strains

BP, a negatively charged nanosheet, is expected to better interact with positively charged cells including Mcr-1 modified *E. coli* cells [8]. Therefore, we expect NZB binding to Mcr-1 modified *E. coli* cells to have increased synergy to PolB, a positively charged antibiotic. To verify our hypothesis, the synergistic activity of NZB was evaluated using checkerboard assays between PolB and NZB with *E. coli* strains BW25113, BW25113 of pQE60-*mcr-1*, NCCP isolates, and NDM-1 MDR strains. According to FICI values (Table 1), NZB to PolB exhibited maximum synergistic activity (FICI_NZB/PolB_ = 0.275–0.3) against *mcr-1* expressing *E. coli* strains, and not for other strains including NDM-1 (FICI _NZB/PolB_ = 2). Therefore, the synergy of NZB to PolB is specific to Mcr-1 modified *E. coli* strains.

It is worthy to note that the NZB and PolB combination has also been evaluated against other Gram-negative strains such as *Pseudomonas aeruginosa* (ATCC27853) (Figure S5a) and *Acinetobacter baumannii* (ATCC19606) (Figure S5b), and a Mcr-1 expressing clinical isolate of *Klebsiella pneumoniae* (NCCP16285) (Figure S5c), where no synergistic activity was observed. This indicates that NZB and PolB combination is specific to only *E. coli* strains expressing Mcr-1, instead of the synergistic action on all Gram-negative strains whenever the expression of Mcr-1 is presented. However, this conclusion should be further validated by the assessment of various bacterial strains with different features.

### 3.3. Morphological Characterization of Bacteria

**Figure 3:**
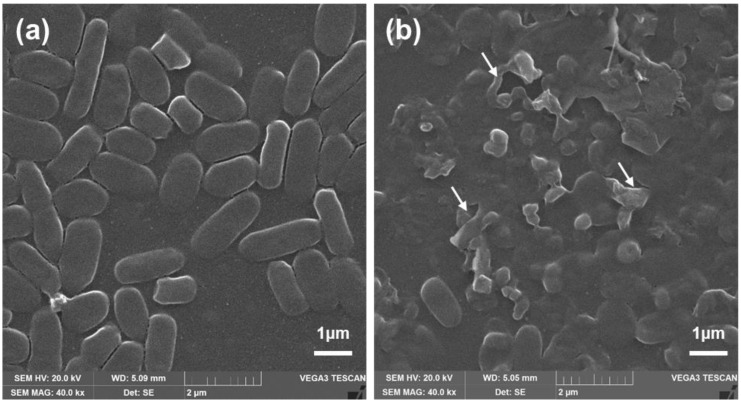
Morphology change of NCCP16283. SEM images (40.0 kx magnification) of NCCP16283 after (**a**) non-treatment and treatment of (**b**) PolB (0.2 μg·mL^−1^) with NZB (62.5 μg·mL^−1^). Images are one of the representatives from *n* = 8. White arrows indicate the disruption of bacterial cells.

For antibacterial properties of PolB, NZO, NZB, and NZB/PolB combination, morphological analysis of bacteria (NCCP16283) under different treatments were performed using SEM. As illustrated in Figure 3a, untreated *E. coli* cells showed typical bacterial morphology with a smooth surface and normal length. Meanwhile, bacterial cells treated with NZB/PolB combination (Figure 3b) showed cell membrane disruption. in particular, the shape of NCCP16283 largely deviated from that of normal cells when treated with NZB/PolB. This indicates that NZB showed a different mechanism of action in bacterial killing and the synergistic action of NZB to PolB is shown (Table 1) to kill more bacterial populations. 

### 3.4. Plausible Mechanism of NZB: Neutralizing Charge of Bacterial Surface

It has been known that polymyxin resistance principally arises from structural remodeling of lipid A anchored on the bacterial surface [7]. In Mcr-1 strains, ArnT and EptA modifications were observed [25]. We questioned whether either of ArnT or EptA modification is required for the synergistic action of NZB to PolB. To test this hypothesis, we performed checkerboard assays of NZB with PolB using null mutants (Keio collections [30]) of *arnT* or *eptA*. Results showed that MICs of PolB and NZB by monotherapy against Keio-*arnT* and -*eptA* strains were the same MIC values as those of BW25113 (MIC = 0.8 and 250 μg·mL^−1^, respectively; Table 1 and Figure S6). However, NZB with PolB against Keio-*arnT* and -*eptA* strains showed synergistic action as in NCCP16283, which contain a Mcr-1 expressing plasmid. This indicates that depletion of either ArnT or EptA expression, which is modified by Mcr-1, is adequate to show the synergistic action of NZB to PolB against *E. coli* cells similar to Mcr-1 clinical isolates.

**Figure 4:**
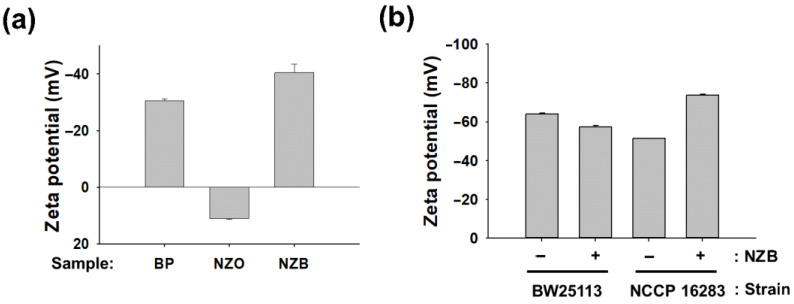
Charge determination of nanomaterials and *Escherichia coli* cells. Zeta potential measurements of (**a**) BP, NZO, and NZB; (**b**) *E. coli* cells of BW25113 and NCCP16283 with or without NZB treatment. BP, NZO, and NZB concentration were set to 125 μg·mL^−1^. Results represent the mean of *n* = 5 ± standard deviation (*p* < 0.05 indicates significant differences).

*E. coli* is generally known to have negative charge on its surface due to the negatively charged lipid A on the LPS component [7,25]. However, Mcr-1 modification changes the charge of *E. coli* cells from negative to marginally positive owing to the lipid A modifications by ArnT and EptA [7,25]. As NZB is a negatively charged nanocomposite, it is expected that charge neutralization between NZB and Mcr-1 modified *E. coli* cells makes cells more prone to attack by a positively charged PolB. To verify our hypothesis, zeta potentials of BP, NZO, and NZB samples were measured. As shown in Figure 4a, values for BP, NZO, and NZB were −30.8, +11.2, and −41.2 mV, respectively. The increase of negatively charged BP in NZB was observed in comparison to BP itself by incorporation of NZO, even though NZO showed a positive charge. This increase in negative charge was shown in a previous report, in which Li^+^ incorporation increased the negative charge of BP [22]. 

We further investigated whether charge on the surface of *E. coli* BW25113 and NCCP16283 is modified by NZB treatment with a sublethal concentration (125 μg·mL^−1^). As shown in Figure 4b, the zeta potential of NZB combination with BW25113 and NCCP16283 was −56.9 and −73.7 mV, respectively. Meanwhile, zeta potential of BW25113 and NCCP16283 without NZB was −63.9 and −51.4 mV, respectively. The results indicate that cells with more negative charge on the surface are less prone to interact with negatively charged NZB. Therefore, cells modified by Mcr-1 showed a decreased negative charge on their surface and may interact more with NZB, which is detrimental to the cell surface membrane as shown in Figure 3b. In this condition, it is much easier for PolB, which is well known as positively charged antibiotic, to bind to the cell membrane of NZB treated *E. coli* cells, making *E. coli* more prone to show synergistic activity to PolB. 

### 3.5. Reactive Oxygen Species (ROS) Production

**Figure 5:**
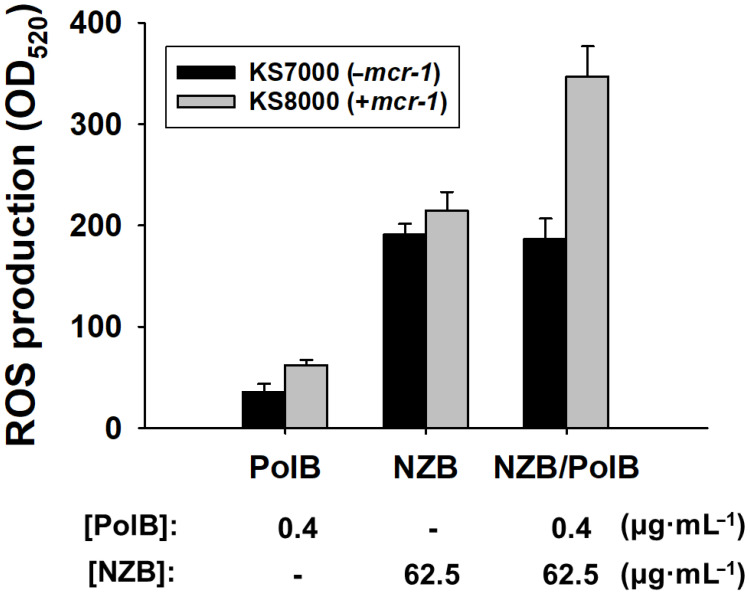
Quantification of ROS production. Fluorescence intensities at 520 nm for *E. coli* KS7000 (−*mcr-1*) and KS8000 (+*mcr-1*) cells treated with PolB, NZB, and NZB/PolB, respectively, were measured. Bacterial cell suspension in PBS without NP treatment was used as a blank. MARS Data Analysis software (ver. 3.02 R2; BMG Labtech GmbH, Ortenber, Germany) was used for data processing and averaged values of relative ROS production by NPs from triplicate experiments were shown (*p* < 0.05).

While SEM images have shown bacterial damage by NZB/PolB combination (Figure 3), it is not clear what particular mechanism is involved in the bacterial cell death by NZB. The production of reactive oxygen species (ROS) is considered to be one of the well-established and efficient antibacterial mechanisms [17]. As an initial mechanistic study of synergistic action of NZB with PolB, ROS production of KS7000 (−*mcr-1*) and KS8000 (+*mcr-1*) cells upon treatment of PolB (0.4 μg·mL^−1^), NZB (62.5 μg·mL^−1^), or NZB (62.5 μg·mL^−1^)/PolB (0.4 μg·mL^−1^), respectively, was evaluated. As shown in Figure 5, both cells produced 1.5 times more ROS upon NZB treatment than that of PolB without preference. However, the combined use of NZB and PolB showed that KS8000 selectively produced 1.5 times more ROS, while KS7000 did not produce more ROS. Therefore, the synergistic antibacterial activity of NZB/PolB combination for Mcr-1 modified *E. coli* cells can be attributed to higher ROS production. 

### 3.6. Biocompatibility of NZB

To determine the lowest concentration for expressing synergy between NZB and PolB, we performed additional checkerboard assays with different ranges of NZB with PolB (Figure S2) and determined that 6.25 or 0.8 μg·mL^−1^ of NZB or PolB, respectively, is the lowest concentration showing FICI_NZB_/_PolB_ = 0.275.

**Figure 6:**
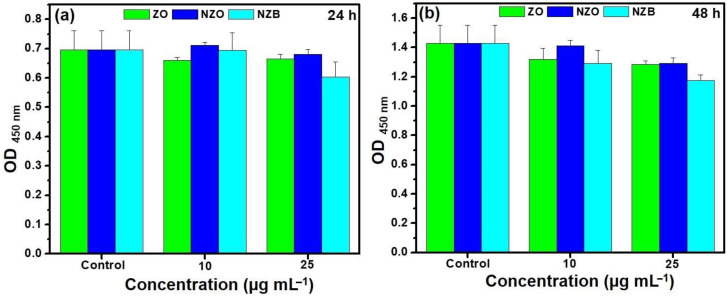
Cell viability (WST assay) of ZO, NZO, and NZB exposure observed in HEK293 (human embryonic kidney) cells with various concentrations for (**a**) 24 h and (**b**) 48 h. Error bars represent the standard deviation (SD). *p* < 0.05 indicates significant differences.

Biocompatibility of nanoparticles is one of the key criteria for its further use in clinical trials [36]. The ability of ZnO NP penetration to kidney is warranted [37]. In addition, the cellular interactions of ZnO NPs with human embryonic kidney (HEK 293) cells has been well defined [38]. Therefore, we assessed in vitro cytotoxicity using WST assays with HEK293 cells at varying concentrations of NZB, a ZnO-based NP, was assessed. As shown in Figure 6, it can be clearly observed that cell viability of the lowest synergistic concentration of NZB, 6.25 μg·mL^−1^, is non-toxic. Therefore, the synergistic concentration of the NZB nanocomposite with PolB is biocompatible and could be effective in the treatment application of polymyxin-resistant bacterial infections. 

## 4. Conclusions

In summary, we have successfully prepared a negatively charged nano-antibacterial NZB using an NZO and BP nanosheet by simple low temperature synthesis and characterized the nanocomposite by XRD, XPS, and TEM. The charge of nanocomposite was analyzed by the zeta potential. Antibacterial activity of NZB and its synergistic activity with PolB against polymyxin-resistant *E. coli* were characterized by checkerboard assays, FICI calculation, and morphological assessment by SEM image analysis. The plausible mechanism of synergy of NZB with PolB specific toward polymyxin-resistant *E. coli* strains was verified by zeta potential analysis. The NZB at the synergistic concentration was also biocompatible. Although it still needs to fully elucidate the mechanism of specific synergistic action of NZB with PolB against *E. coli*, our results firstly showed that charge neutralizing NPs could be synergistic nanomaterials potentially used for further clinical applications including therapeutics and specific detection of polymyxin-resistant *E. coli*. This is a cost-effective approach to develop a new generation of last resort antibiotics, which are now considered to be out of the market. However, considering that ZnO NP as a cytotoxic material when it is exposed to mammals with higher concentrations for a long time, it is still challenging to design and synthesize biodegradable NZBs that do not accumulate over non-toxic concentration in the kidney.

## Figures and Tables

**Table 1:** MIC (Minimum inhibitory concentration) and FICI (Fraction inhibitory concentration index) values for NZB, PolB, and NZB/PolB against Gram-negative strains ^1^.

Strain	Presence of *mcr-1* ^2^	Monotherapy		Synergistic Combination				
		MIC (μg·mL^−1^)		MIC (μg·mL^−1^)				Maximum FICI_NZB/PolB_
		PolB	NZB	PolB	Fold Change to Monotherapy	NZB	Fold Change to Monotherapy	
	***E. coli*** **strains**							
BW25113	−	0.8	250	0.8	1	250	1	2
Keio-*arnT*	−	0.8	250	0.2	4	31.25	8	0.375
Keio-*eptA*	−	0.8	250	0.2	4	31.25	8	0.375
NCCP16283	+	3.2	250	0.8	4	12.5	20	0.3
NCCP16284	+	3.2	250	0.8	4	12.5	20	0.3
BAA-2471	−	0.8	250	0.4	2	125	2	1
BAA-2340	−	0.8	250	0.4	2	125	2	1
KS7000 ^3^	−	0.8	250	0.4	2	125	2	1
KS8000 ^4^	+	6.4	250	1.6	4	6.25	40	0.275
	**Non-*E. coli* Gram-negative strains**							
NCCP16285	+	6.4	250	6.4	1	>250	>1	>2
ATCC19606	−	3.2	>250	3.2	1	>250	1	2
ATCC27853	−	3.2	>250	3.2	1	>250	1	2

^1^ Values were obtained from the calculations as described in Ansari et al. [31] using Figures S1, S2, S5, and S6. ^2^ Presence of Mcr-1 expressing plasmid was indicated. + and − indicate that either Mcr-1 expressing plasmid was presented or not presented, respectively. ^3^ KS7000 or ^4^ KS8000 contain pQE60 or pQE60-*mcr-1* plasmid in BW25113 strain, respectively (Table S2). Abbreviations: PolB: polymyxin B, NZB: nickel doped zinc oxide (ZnO) with black phosphorous, FICI: fractional inhibitory concentration index/indices, NDM: New Delhi metallo-β-lactamase-1, *mcr-1*: mobilized colistin resistance.

## Data Availability

Data sharing is not applicable to this article.

## Supplementary Materials

The following are available online at https://www.mdpi.com/1999-4923/13/2/250/s1, Table S1: Sequence of synthesized mobilized colistin resistance gene (*mcr-1*), Table S2: Strains and plasmids used in this study, Figure S1: Synergy activity of NZB to polymyxin B (PolB) against *E. coli* cells, Figure S2: Synergistic activity of NZB to polymyxin B (PolB) against Mcr-1 expressing *E. coli* cells, Figure S3: Features of Mcr-1 protein expression plasmid (pQE60-*mcr-1*), Figure S4: Expression of Mcr-1 protein, Figure S5: Synergistic activity of NZB to polymyxin B (PolB) against non-*E. coli* Gram-negative strains, Figure S6: Evaluation of synergistic activity of Mcr-1 modified gene knockouts.
