# Supplementary material for: A New Surface Charge Neutralizing Nano-Adjuvant to Potentiate Polymyxins in Killing Mcr-1 Mediated Drug-Resistant Escherichia coli
Source: Pharmaceutics. 2021 Feb 11;13(2):250. doi: 10.3390/pharmaceutics13020250 (PMC7917812; doi:10.3390/pharmaceutics13020250)
Supplement: Supplementary file 1 [file pharmaceutics-13-00250-s001.pdf]

pharmaceutics

MDPI

Supplementary Materials: A New Surface Charge Neutralizing Nano-Adjuvant to Potentiate Polymyxins in Killing Mcr-1 Mediated Drug-Resistant *Escherichia coli*

Hyejin Cho, Atanu Naskar, Sohee Lee, Semi Kim and Kwang-sun Kim

Table S1. Sequence of synthesized mobilized colistin resistance gene (*mcr-1*). The sequences of *mcr-1* were taken from NCBI protein database (GenBank ID: ASK04346.1) and further codon optimized by GenSmart™ Codon Optimization (ver. Beta 1.0; https://www.genscript.com/tools/gensmart-codon-optimization; GenScript, Piscataway, NJ, USA). Red sequences indicate the modified DNA sequences by codon optimization.

Gene	DNA Sequence (5′ → 3′)
***mcr-1* (1627 bp)**	ATGATGCAGCACACCAGCGTTTGGTATCGTCGTAGCGTTAGCCCGTTCGTTCTGGTTGCG<br>AGCGTTGCGGTTTTCCTGACCGCGACCGCGAACCTGACCTTCTTTGACAAGATCAGCCAA<br>ACCTACCCGATTGCGGATAACCTGGGTTTCGTTCTGACCATCGCGGTGGTTCTGTTTGGC<br>GCGATGCTGCTGATTACCACCCTGCTGAGCAGCTACCGTTATGTGCTGAAACCGGTTCTG<br>ATCCTGCTGCTGATTATGGGTGCGGTTACCAGCTACTTCACCGACACCTACGGCACCGTG<br>TATGATACCACCATGCTGCAGAACGCGCTGCAGACCGACCAAGCGGAAACCAAGGATCTG<br>CTGAACGCGGCGTTCATTATGCGTATCATTGGTCTGGGCGTGCTGCCGAGCCTGCTGGTT<br>GCGTTTGTGAAGGTTGACTACCCGACCTGGGGTAAAGGCCTGATGCGTCGTCTGGGTCTG<br>ATCGTGGCGAGCCTGGCGCTGATTCTGCTGCCGGTGGTTGCGTTCAGCAGCCACTATGCG<br>AGCTTCTTTCGTGTGCACAAGCCGCTGCGTAGCTACGTTAACCCGATCATGCCGATTTAT<br>AGCGTTGGCAAACTGGCGAGCATCGAATACAAGAAAGCGAGCGCGCCGAAGGACACCATT<br>TATCACGCGAAAGATGCGGTGCAAGCGACCAAGCCGGACATGCGTAAACCGCGTCTGGTG<br>GTTTTTGTGGTTGGTGAAACCGCGCGTGCGGATCACGTTAGCTTCAACGGCTACGAACGT<br>GACACCTTTCCGCAGCTGGCGAAGATCGATGGTGTGACCAACTTCAGCAACGTTACCAGC<br>TGCGGTACCAGCACCGCGTACAGCGTGCCGTGCATGTTTAGCTACCTGGGTGCGGATGAG<br>TATGACGTGGATACCGCGAAATATCAAGAAAACGTTCTGGACACCCTGGATCGTCTGGGT<br>GTGAGCATCCTGTGGCGTGACAACAACAGCGATAGCAAGGGCGTTATGGACAAGCTGCCG<br>AAAGCGCAGTTCGCGGATTACAAAAGCGCGACCAACAACGCGATTTGCAACACCAACCCG<br>TATAACGAGTGCCGTGACGTGGGTATGCTGGTTGGCCTGGACGATTTCGTTGCGGCGAAC<br>AACGGCAAGGATATGCTGATCATGCTGCACCAAATGGGTAACCACGGCCCGGCGTACTTT<br>AAACGTTATGACGAGAAGTTCGCGAAATTTACCCCGGTGTGCGAGGGCAACGAACTGGCG<br>AAATGCGAACACCAGAGCCTGATCAACGCGTACGATAACGCGCTGCTGGCGACCGACGAT<br>TTCATCGCGCAGAGCATTCAATGGCTGCAGACCCACAGCAACGCGTACGACGTGAGCATG<br>CTGTATGTTAGCGATCACGGCGAGAGCCTGGGTGAAAACGGCGTTTATCTGCACGGCATG<br>CCGAACGCGTTTGCGCCGAAGGAACAACGTAGCGTGCCGGCGTTCTTTTGGACCGACAAA<br>CAGACCGGTATCACCCCGATGGCGACCGACACCGTGCTGACCCACGATGCGATTACCCCG<br>ACCCTGCTGAAGCTGTTTGATGTTACCGCGGACAAGGTGAAGGACCGTACCGCGTTTATT<br>CGT

Table S2. Strains and plasmids used in this study.

Name	Feature	Reference[1]
**Strains**		
BW25113	*F⁻Δ(araD-araB)567*, *ΔlacZ4787*::*rrnB-3*, *LAM⁻ rph-1 Δ(rhaD-rhaB)568 hsdR514*	[1]
Keio-*arnT*	BW25113*ΔarnT*::kan^R	[2]
Keio-*eptA*	BW25113*ΔeptA*::kan^R	[2]
KS7000	BW25113 pQE60	This study
KS8000	BW25113 pQE60-*mcr-1*	This study
ATCC19606	*Acinetobacter baumannii* Bouvet and Grimont	American Type Culture Collection (ATCC) (www.atcc.org)
ATCC27853	*Pseudomonas aeruginosa* (Schroeter) Mugula	ATCC
**Clinical isolates**		
NCCP16283	*Escherichia coli*, Colistin^R, *mcr-1*	National Culture Collections for Pathogens (NCCP)
NCCP16284	*Escherichia coli*, Colistin^R, *mcr-1*, *bla*$_{NDM-1}$, ESBLs	NCCP
NCCP16285	*Klebsiella pneumoniae*, Colistin^R, *mcr-1*, *bla*$_{NDM-1}$, ESBLs	NCCP
BAA-2340	Carbapenems^R, *bla*$_{NDM}$-/*bla*$_{KPC}$+	ATCC
BAA-2471	Carbapenems^R, bla$_{NDM}$+/*bla*$_{KPC}$-	ATCC
**Plasmids**		
pQE60	Ampicillin	Qiagen
pQE60-*mcr-1*	Colistin, polymyxin B, Ampicillin	This study
**Primer sequence (5′ → 3′)**		
pQE60-F	CCC GAA AAG TGC CAC CTG	
pQE60-R	GTT CTG AGG TCA TTA CTG G	

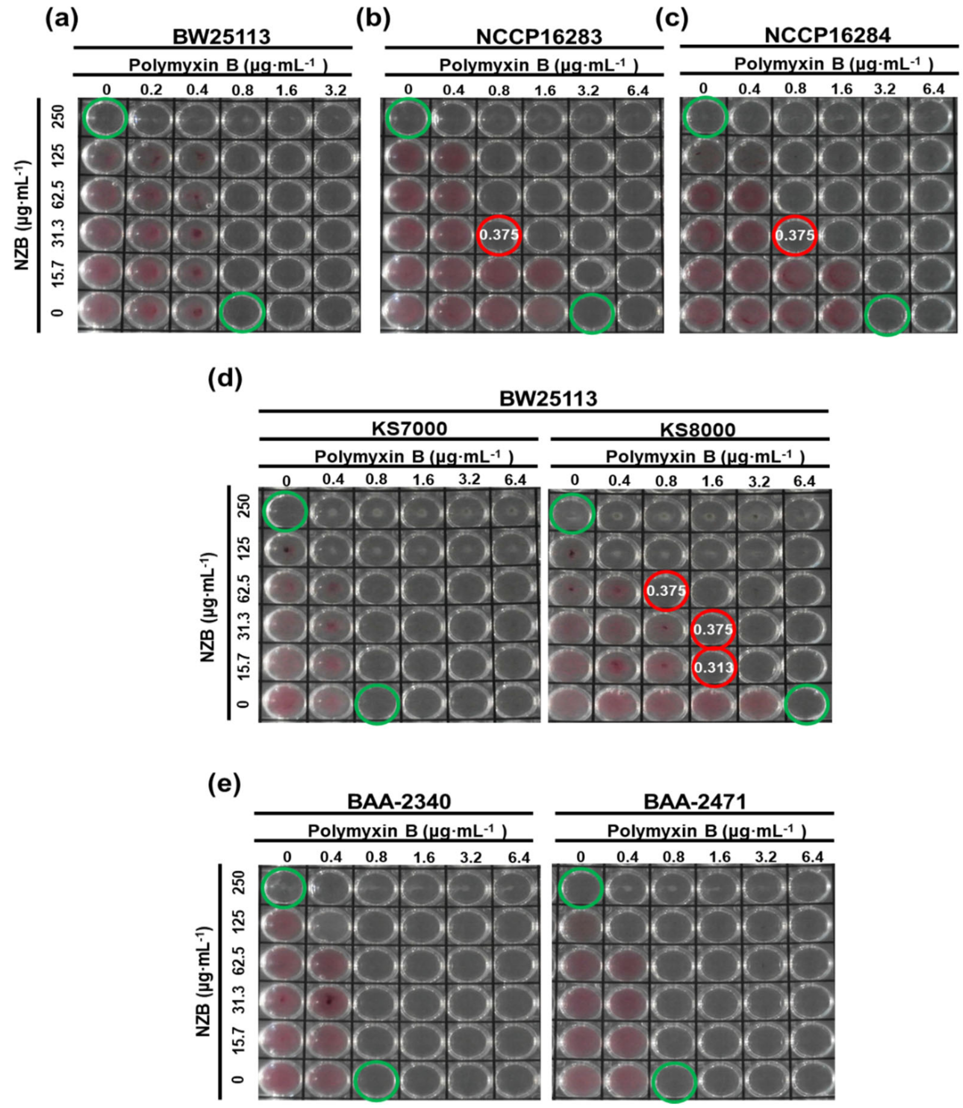

Figure S1. Synergy activity of NZB to polymyxin B (PolB) against *E. coli* cells. Checkerboard assays for (**a**) BW25113 and (**b**)–(**d**) *mcr-1* harboring *E. coli* cells either plasmid incorporated (pQE60 or pQE60-*mcr-1* in BW25113, i.e. KS7000 or KS8000, respectively) or clinical isolates (NCCP16283 and 16284), (**e**) MDR *E. coli* cells (BAA-2340 and -2471) were shown. The MIC point of NZB and PolB was shown in green circle. Synergistic positions (FICI < 0.5) were indicated with red circles. One of the representatives from n = 3 was shown. FICI values were indicated in Table 1. The strain information is available in Table S1.

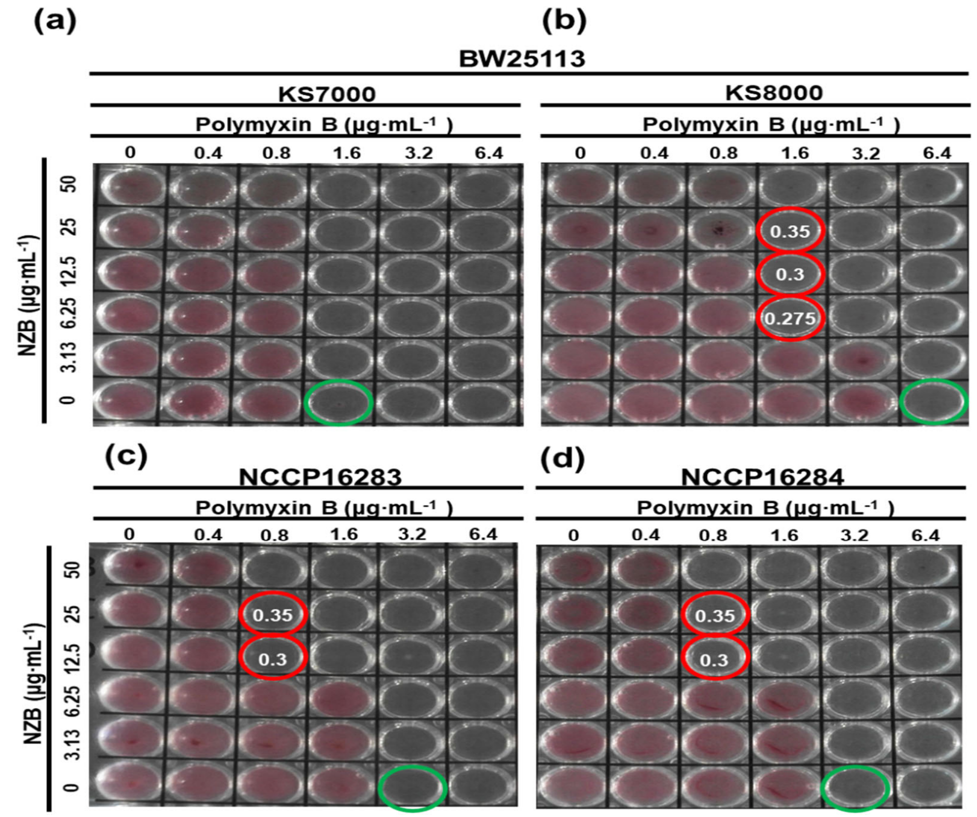

Figure S2. Synergistic activity of NZB to polymyxin B (PolB) against Mcr-1 expressing *E. coli* cells. Checkerboard assays results for (**a**) KS7000 (pQE60 in BW25113; non-*mcr-1*), (**b**) KS8000 (pQE60-*mcr-1* in BW25113), (**c**) NCCP16283, and (**d**) NCCP16284 were shown. The MIC point of PolB was shown in green circle. Synergistic positions (FICI < 0.5) were indicated with red circles. One of the representatives from n = 3 was shown. The stain information is available in Table S2. FICI values were shown in Table 1.

(a)

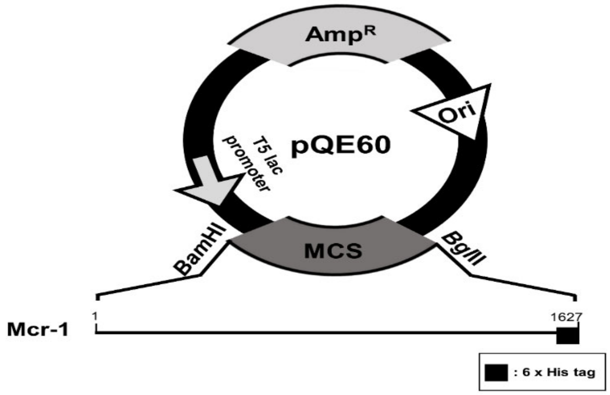

(b)

Protein	Amino acid sequence
Mcr-1	MMQHTSVWYRRSVSPFVLVASVAVFLTATANLTFFDKISQTYPIADNLGFV<br>LTIAVVLFGAMLLITTLLSSYRYVLKPVLILLLIMGAVTSYFTDTYGTVYDTTM<br>LQNALQTDQAETKDLLNAAFIMRIIGLGVLPSLLVAFVKVDYPTWGKGLMR<br>RLGLIVASLALILLPVVAFSSHYASFFRVHKPLRSYVNPIMPIYSVGKLASIE<br>YKKASAPKDTIYHAKDAVQATKPDMRKPRLVVFVVGETARADHVSFNGYE<br>RDTFPQLAKIDGVTNFSNVTSCGTSTAYSVPCMFSYLGADEYDVDTAKYQ<br>ENVLDTLDRLGVSILWRDNNSDSKGVMDKLPKAQFADYKSATNNAICNTN<br>PYNECRDVGMLVGLDDFVAANNGKDMLIMLHQMGNHGPAYFKRYDEKFA<br>KFTPVCEGNELAKCEHQSLINAYDNALLATDDFIAQSIQWLQTHSNAYDVS<br>MLYVSDHGESLGENGVYLHGMPNAFAPKEQRSVPAFFWTDKQTGITPMA<br>TDTVLTHDAITPTLLKLFDVTADKVKDRTAFIR

Figure S3. Features of Mcr-1 protein expression plasmid (pQE60-*mcr-1*). (**a**) Schematic representation of Mcr-1 protein coding regions with C-terminal histidine tag in pQE60 vector and (**b**) amino acid sequences of Mcr-1.

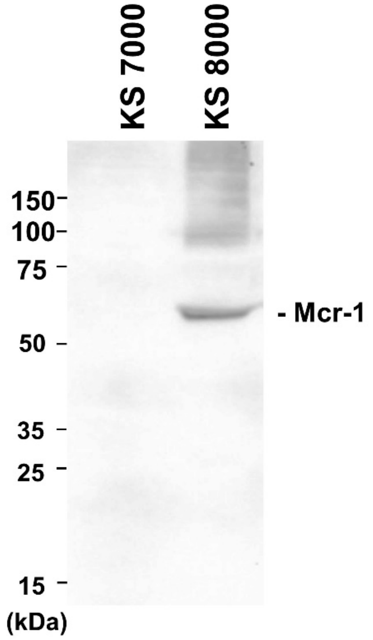

Figure S4. Expression of Mcr-1 protein. Mcr-1 expression from KS7000 (pQE60) and KS8000 (pQE60-*mcr-1*) strains was detected by western blotting with antibodies against His-tag. Image acquisition and the quantitative analysis were performed by using the ChemiDoc MP Imaging System (Bio-Rad, Hercules, CA, USA) and Image Lab (ver 5.2.1; Bio-Rad, Hercules, CA, USA).

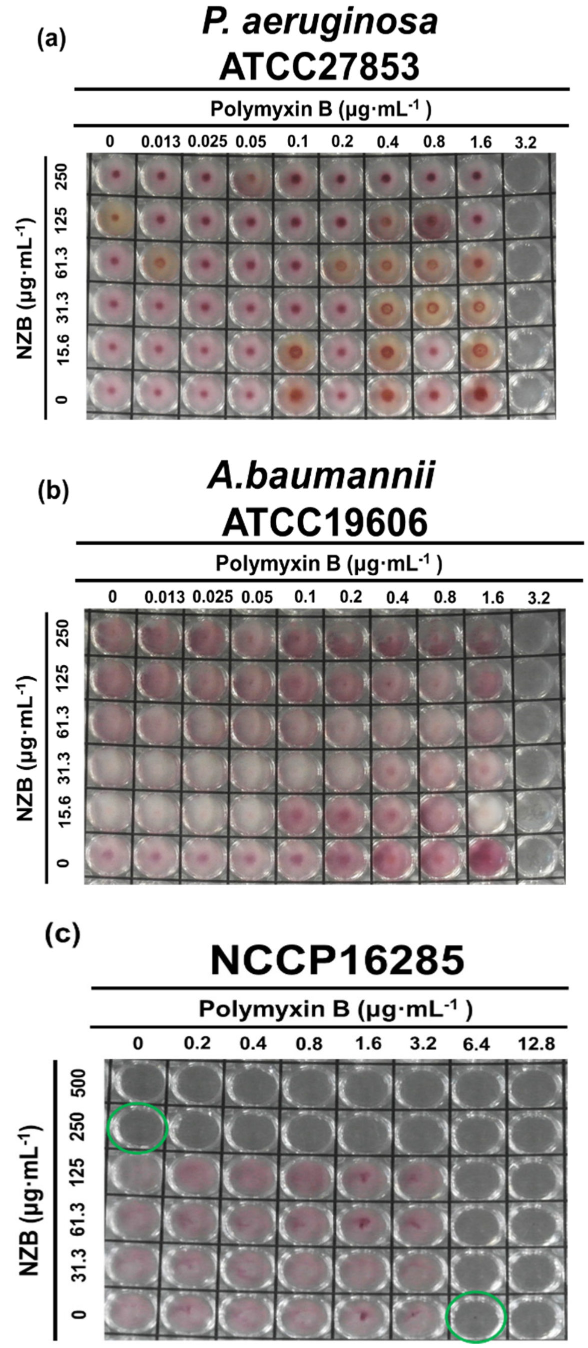

Figure S5. Synergistic activity of NZB to polymyxin B (PolB) against non-*E. coli* Gram-negative strains. Checkerboard assays results for (**a**) ATCC27853 (*P. aeruginosa*), (**b**) ATCC19606 (*A. baumannii*), and (**c**) NCCP16285 (*Klebsiella pneumoniae mcr-1* clinical isolates) were shown. One of the representatives from $n = 3$ was shown. The MIC point of PolB was shown in green circle. Synergistic positions (FICI < 0.5) were indicated with red circles. FICI values were indicated in Table 1.

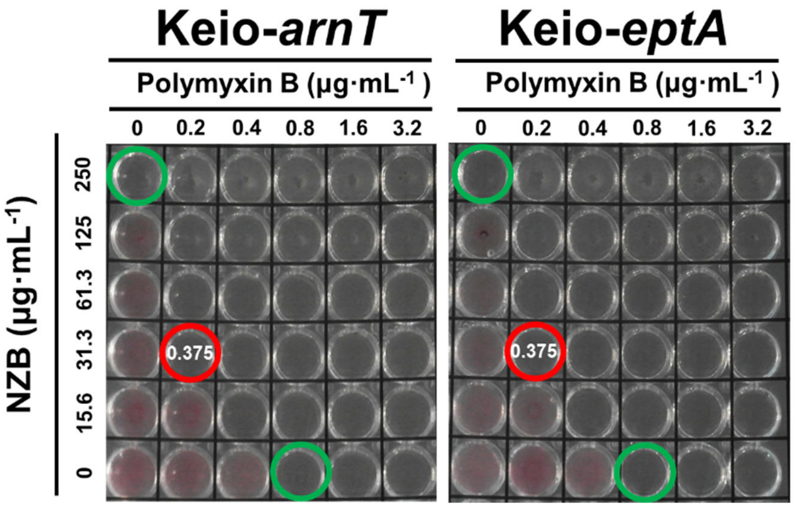

Figure S6. Evaluation of synergistic activity of Mcr-1 modified gene knockouts. Checkerboard assays for NZB and polymyxin B (PolB) against Keio-*arnT* (left) and Keio-*eptA* (right) were shown. One of the representatives from $n = 3$ was shown. The MIC point of PolB was shown in green circle. Synergistic positions (FICI < 0.5) were indicated with red circles. FICI values were shown in Table 1.

References

1. Datsenko, K.A.; Wanner, B.L. One-step inactivation of chromosomal genes in *Escherichia coli* K-12 using PCR products. *Proc. Natl. Acad. Sci. USA*. **2000**, *97*, 6640–6645.
2. Baba, T.; Ara, T.; Hasegawa, M.; Takai, Y.; Okumura, Y.; Baba, M.; Datsenko, K.A.; Tomita, M.; Wanner, B.L.; Mori, H. Construction of *Escherichia coli* K-12 in-frame, single-gene knockout mutants: the Keio collection. *Mol. Syst. Biol*. **2006**, *2*, 2006.0008.
